# Interplay between hopping dimerization and quasi-periodicity on flux-driven circular current in an incommensurate Su–Schrieffer–Heeger ring

**DOI:** 10.1038/s41598-023-31354-9

**Published:** 2023-03-11

**Authors:** Souvik Roy, Sudin Ganguly, Santanu K. Maiti

**Affiliations:** 1 Physics and Applied Mathematics Unit, Indian Statistical Institute, 203 Barrackpore Trunk Road, Kolkata, 700108 India; 2 Department of Physics, School of Applied Sciences, University of Science and Technology Meghalaya, Ri-Bhoi, 793101 India

**Keywords:** Nanoscience and technology, Physics

## Abstract

We report for the first time the phenomenon of flux-driven circular current in an isolated Su–Schrieffer–Heeger (SSH) quantum ring in presence of cosine modulation in the form of the Aubry–André–Harper (AAH) model. The quantum ring is described within a tight-binding framework, where the effect of magnetic flux is incorporated through Peierls substitution. Depending on the arrangements of AAH site potentials we have two different kinds of ring systems that are referred to as staggered and non-staggered AAH SSH rings. The interplay between the hopping dimerization and quasiperiodic modulation leads to several new features in the energy band spectrum and persistent current which we investigate critically. An atypical enhancement of current with increasing AAH modulation strength is obtained that gives a clear signature of transition from a low conducting phase to a high conducting one. The specific roles of AAH phase, magnetic flux, electron filling, intra- and inter-cell hopping integrals, and ring size are discussed thoroughly. We also study the effect of random disorder on persistent current with hopping dimerization to compare the results with the uncorrelated ones. Our analysis can be extended further in studying magnetic responses of similar kinds of other hybrid systems in presence of magnetic flux.

## Introduction

The localization phenomenon has always been an active field of research^1–5^ in the discipline of condensed matter physics since its prediction by P. W. Anderson in 1958. The interest in this topic gets increased day by day with the development of different kinds of fascinating systems, spanning over a wide branch of physics. In his pioneering work^1^, Anderson showed that for a one-dimensional atomic system with ‘uncorrelated’ site energies, all the energy eigenstates are completely localized irrespective of the disorder strength. Such a system is rather quite trivial, as on one hand the critical disorder strength $W_{c}$ is zero (*W* measured the strength of disorder), and on the other hand, we do not have any specific control over it. Interesting behavior arises when a restriction is imposed on the site energies, to make the system a ‘correlated’ disordered one^6–10^. So far, various kinds of correlated systems have been used in different areas, and among many, the most common and versatile example of such a system is referred to as Aubry–André–Harper (AAH) model^11–21^.

For a one-dimensional tight-binding (TB) AAH chain with nearest-neighbor hopping (NNH) integral *t*, it is well established that there exists a sharp transition at a finite critical point $W_{c}=2t$^11,13,14^. For $W<W_{c}$, all the states are fully conducting, while they become absolutely localized when $W>W_{c}$. At $W=W_{c}$, critical states are obtained. In this case, the mixing of conducting and localized states is not available, and thus, *mobility edge* does not appear^13,14^. The existence of non-zero critical point leads to different atypical signatures in physical properties and during the last several years an enormous amount of work has been done in different areas starting from electron transport, spin selective transport phenomena, controlled light propagation, and to name a few^22–25^.

Now, a basic question may arise about how a physical system behaves when a further restriction is given which is already subjected to an AAH type of modulation. The additional restriction can be imposed with the inclusion of dimerized hopping integrals. The one-dimensional lattice with such hopping integrals is the simplest example of a non-trivial topological system and is well-known as the Su–Schrieffer–Heeger (SSH) model^26–30^. Individually the systems with AAH modulation, and, the SSH lattices have been investigated extensively, but studies considering both these effects are relatively scarce. Motivated by these facts, in the present paper we explore the interplay between the hopping dimerization and quasi-periodicity on electronic localization and on a specific physical phenomenon which is the magnetic response of non-interacting electrons in a mesoscopic ring in presence of a magnetic flux $\phi$. The inclusion of magnetic flux in an isolated conducting mesoscopic ring generates a circular current, and once this current is established it never vanishes even when the flux gets removed. Such a fascinating magnetic response is referred to as flux-driven persistent current and was first proposed more than three decades ago by Büttiker, Imry, and Landauer^31^. After this proposal, a substantial amount of theoretical and experimental work has been done^32–47^, and a wealth of literature knowledge has already been developed on this topic. But no attempt has been made so far considering an AAH SSH ring. As the flux-driven circular current is directly involved with the variation of energy levels with flux $\phi$, from the analysis of energy band spectrum and persistent current, the combined effect of AAH modulation of dimerized hopping integrals can be well understood.

**Figure 1:**
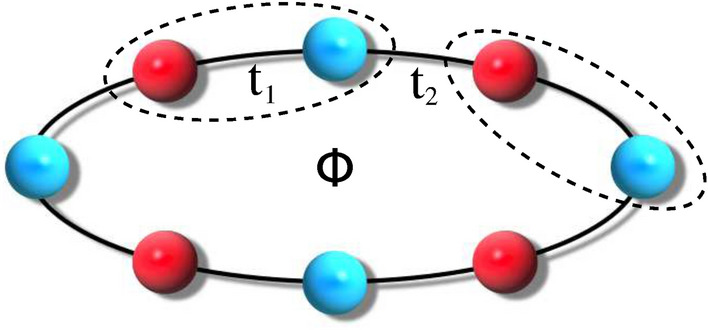
AAH ring with dimerized hopping integrals, threaded by a magnetic flux $\phi$.

We describe the AAH SSH ring within a tight-binding framework and include the effect of magnetic flux, commonly referred to Aharonov–Bohm (AB) flux^48,49^, through Peierls substitution. Two different kinds of NNH integrals, $t_{1}$ and $t_{2}$, are taken into account (see Fig. 1), like what is used in the 1D SSH model, and depending on the specific arrangement of AAH site potentials we have two different configurations that are referred to as ‘non-staggered’ and ‘staggered’ AAH SSH rings. In this communication, we critically investigate the critical roles played by $t_{1}$ and $t_{2}$ in both these cases. Determining the energy eigenvalues of the TB Hamiltonian matrix of the ring system, we compute flux-dependent circular current using the well-known derivative method^32,41^ under different input conditions. For the sake of completeness, we also study the behavior of persistent current in the presence of random disorder in SSH rings. The key findings that emerge from our detailed numerical analysis are as follows. (1) The energy band structure gets significantly modified. A reasonable enhancement of the slopes of some energy levels with respect to $\phi$ occurs with the increase of the AAH modulation strength. It is directly reflected in the current spectrum. A clear indication of transition from a low conducting phase to a high conducting one is seen, which is in complete contrast to the literature knowledge. (2) The current amplitude can be monitored selectively by regulating the phase factor associated with the AAH modulation, keeping all the other parameters constant. (3) Filling factor plays an important role to get the circular current. Both for the staggered and non-staggered cases, the current shows large values near the half-filled band case. (4) The hopping dimerization leads to the co-existence of localized and extended energy levels, though the ring is described with only NNH integrals and this feature is observed irrespective of the nature of the disorderedness, that is correlated (AAH) or uncorrelated (random). The localization behavior of any energy eigenstate is described in terms of the inverse participation ratio (IPR)^50–56^. Our analysis may provide a new route of expecting anomalous physical phenomena in such hybrid systems where both AAH and SSH effects are included.

With the above brief introduction in “1Introduction” section, In “2Quantum ring, tight-binding Hamiltonian and theoretical prescription” section we present the ring geometry, its Hamiltonian, and the theoretical prescription for calculating the results. All the essential findings are critically discussed in “3Numerical results and discussion” section. Finally, in “4Closing remarks” section, the summary of the work is given.

## Quantum ring, tight-binding Hamiltonian and theoretical prescription

In this section, we illustrate the ring geometry, corresponding Hamiltonian in the tight-binding framework, and the theoretical prescription for calculating energy eigenvalues and flux-driven persistent current.

### Quantum ring and the TB Hamiltonian

The schematic view of the ring geometry that is used to investigate magnetic response in presence of AB flux $\phi$ is shown in Figure 1. The ring is formed by *N* unit cells, where each unit is composed of two different atoms, represented by red and cyan balls. We label these balls as $\alpha$ and $\beta$ sites respectively, for the simplification of the description. The quantum system is simulated within a TB framework and it reads as,

1 $$\begin{aligned} H= & {} \sum _{n} \varepsilon _{\alpha ,n}c_{\alpha ,n}^{\dagger } c_{\alpha ,n}+ \sum _{n} \varepsilon _{\beta ,n}c_{\beta ,n}^{\dagger } c_{\beta ,n}\nonumber \\{} & {} +\sum _{n} \left( t_{1}e^{i\theta }c_{\alpha ,n}^{\dagger } c_{\beta ,n}+ t_{1}e^{-i\theta }c_{\beta ,n}^{\dagger } c_{\alpha ,n}\right) \nonumber \\{} & {} + \sum _{n} \left( t_{2}e^{i\theta }c_{\beta ,n}^{\dagger } c_{\alpha ,n+1}+t_{2}e^{-i\theta }c_{\alpha ,n+1}^{\dagger } c_{\beta ,n}\right) \end{aligned}$$

where *n* is the unit cell index and it runs from 1 to *N*, and $c_{\alpha (\beta ),n}^{\dagger }$, $c_{\alpha (\beta ),n}$ are the usual fermionic operators. $t_{1}$ and $t_{2}$ represent the intra- and inter-cell hopping integrals, respectively. Due to the AB flux $\phi$, a phase factor $\theta$ is introduced in the hopping terms called the AB phase, and it is expressed as^32,41^ $\theta =2 \pi \phi /(2N)$ (2*N* being the total number of sites in the ring). The flux $\phi$ is measured in the unit of the elementary flux-quantum $\phi _{0}$ ($=ch/e$, *c*, *h* and *e* are the fundamental constants). $\varepsilon _{\alpha ,n}$ and $\varepsilon _{\beta ,n}$ are the site energies at the sites $\alpha$ and $\beta$ of *n*th unit cell, respectively. A cosine modulation in the form of the AAH model is introduced in the site energies. We can express them as^12–14^: $\varepsilon _{\alpha ,n}=W_{\alpha }\cos (2\pi b n+\phi _{\nu })$ and $\varepsilon _{\beta ,n}=W_{\beta }\cos (2\pi b n+\phi _{\nu })$, where $W_{\alpha (\beta )}$ is the cosine modulation strength at the site $\alpha (\beta )$. $\phi _{\nu }$ is the phase factor associated with the AAH modulation and it has a special significance as one can tune this factor externally^15^ with the help of a suitable setup. The factor *b* is an irrational number, which leads to the aperiodicity in the lattice. For our numerical calculations, we choose^13,14^ $b=(1+\sqrt{5})/2$, though any other irrational number can also be taken into account, without loss of any generality. Depending on $W_{\alpha }$ and $W_{\beta }$, we have two different configurations, one is referred to as ‘non-staggered’ where $W_{\alpha }=W_{\beta }=W$, and, the other one is called ‘staggered’ ring where $W_{\alpha }=-W_{\beta }=W$. For both these configurations, we thoroughly investigate magnetic responses in different parameter regimes.

Another important point should be mentioned here is that a correlated disordered system is completely different from an uncorrelated or so-called ‘random’ disordered system. While both kinds of disordered systems show localization behavior, uncorrelated disorder has a much stronger effect on localization than a correlated one. In the present work, first, we study the correlated viz., AAH disordered systems and compare the results with the random disordered systems.

### Theoretical formulation

#### Energy eigenvalues, ground state energy, and circular current

The energy eigenvalues $E_{m}$ of the ring geometry are obtained by directly diagonalizing its TB Hamiltonian matrix. Getting the discrete energy eigenvalues, we compute the ground state energy, at absolute zero temperature, when the ring contains $N_{e}$ number of electrons by summing over the lowest $N_{e}$ energy levels, and it becomes^32^

2 $$\begin{aligned} E_{0} (\phi )=\sum _{m} E_{m} (\phi ). \end{aligned}$$

Taking the first order derivative of the ground state energy $E_{0}(\phi )$ with respect to the flux $\phi$, the persistent current $I_{\phi }$ in the ring is obtained. It is defined as^32^

3 $$\begin{aligned} I_{\phi }= -c\frac{\partial E_{0}(\phi )}{\partial \phi } \end{aligned}$$

This is the standard protocol for calculating flux-driven circular current in an AB loop and has been extensively used in the literature. In this method, we do not need to bother explicitly about the energy eigenstates of the system, as the current is obtained from the flux-dependent energy levels.

#### Calculation of IPR

To investigate whether the extended and localized states co-exist or not, and also to check the nature of individual energy eigenstates, we compute inverse participation ratios (IPRs). For any arbitrary eigenstate $|\psi _{p}\rangle$ ($=\sum _{m} a_{m}^{p} |m\rangle$), the IPR is expressed as^50–53^

4 $$\begin{aligned} IPR_{p}=\sum _{m}|a_{m}^{p}|^{4} \end{aligned}$$

where $a_{m}^{p}$’s are the coefficients and $|m\rangle$’s are the Wannier states. The localizing behavior of different states of an isolated system can be easily described by means of IPR. For an extended state, an electron contributes at all the lattice sites^15^ and thus IPR $\rightarrow 0$, while for a localized state, electron gets pinned at a particular lattice site and hence IPR becomes finite^15^. In the asymptotic limit, the maximum value of IPR for a localized state reaches unity, but it will be pretty hard to achieve this limiting value for a ‘finite-size’ system.

## Numerical results and discussion

In what follows we present our numerical results for the AAH SSH quantum ring system under different input conditions, and explore the critical roles played by the hopping dimerization and AAH modulation on energy band spectrum, flux-driven circular current, and electronic localization phenomena. The results are arranged and discussed in different sub-sections. Depending on $t_{1}$ and $t_{2}$, two different cases are taken into account: one is $t_{1}<t_{2}$ and other is $t_{1}>t_{2}$. For both these cases $t_{1}$ is fixed to 1 eV, whereas $t_{2}$ becomes 1.5 eV and 0.5 eV, respectively, depending on the conditions. Unless specified, the AAH phase $\phi _{\nu }$ is set to zero. The other physical parameters are mentioned in the appropriate places.

### Spectral properties

**Figure 2:**
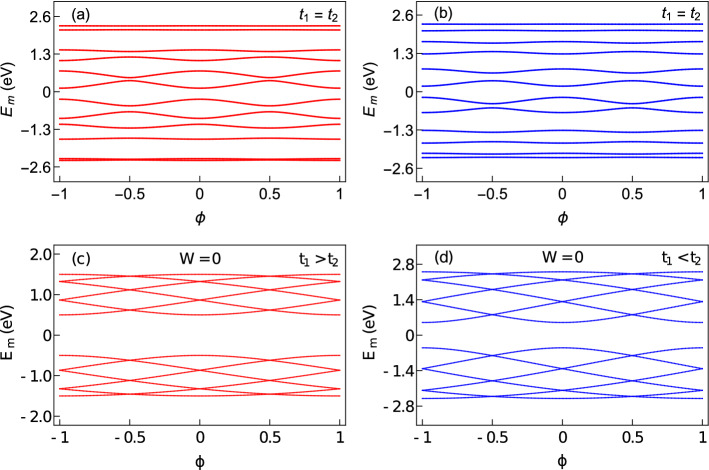
Dependence of energy eigenvalues as a function of AB flux $\phi$. The first row represents the results of the ring subjected to only AAH modulation with $W=1$ and $t_{1}=t_{2}=1$, where (**a**) and (**b**) correspond to non-staggered and staggered cases, respectively. The second row denotes the results of an SSH ring (without any AAH modulation) where (**c**) $t_{1}>t_{2}$ ($t_{1}=1$, $t_{2}=0.5$) and (**d**) $t_{1}<t_{2}$ ($t_{1}=1$, $t_{2}=1.5$). For all these cases, we set $N=6$ and $\phi _{\nu }=0$.

To understand the electronic properties of AAH SSH rings, we first study the behavior of energy eigenvalues with AB flux $\phi$ for the following cases. We first consider non-staggered and staggered AAH rings with no hopping dimerization as shown in Fig. 2a and b, respectively and then pure SSH ring with $t_{1}>t_{2}$ (Fig. 2c) and $t_{1}<t_{2}$ (Fig. 2d). Here we take a relatively smaller ring with $N=6$, such that all the energy levels are seen quite clearly. Usually, for a pure AAH ring, the energy eigenspectrum splits into three bands^41^. That splitting, however, is not prominent for both the non-staggered and staggered AAH rings with AAH modulation strength $W=1$ eV, as is seen from Fig. 2a and b, respectively. In both these cases, apart from the band edges, all the energy levels exhibit finite variations with flux $\phi$, which is the primary condition to have a non-zero flux-driven circular current. On the other hand, the energy spectrum as a function of $\phi$ is broken into two bands for the pure SSH rings (i.e. no AAH) with $t_{1}>t_{2}$ and $t_{1}<t_{2}$ as we observe from Fig. 2c and d, respectively. Such a separation of the whole energy band into two bands is the hallmark of SSH chain due to the hopping dimerization and also seems to be present in the SSH ring. Moreover, the energy levels closer to the center of the band have greater slopes compared to the states located near the band edges. Thus a filling-dependent current is expected to be observed.

**Figure 3:**
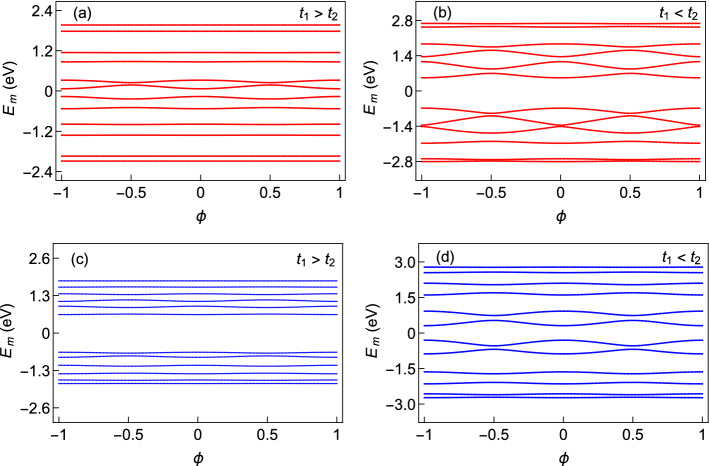
Dependence of energy eigenvalues as a function of AB flux $\phi$ for the AAH SSH rings at AAH disorder strength $W=1$, where first and second rows denote the results for the non-staggered and staggered cases, respectively. The values of *N*, $\phi _{\nu }$, $t_{1}$ and $t_{2}$ for $t_{1}<t_{2}$ and $t_{1}>t_{2}$ remain same as taken in Fig. 2, for a clear comparison of the energy spectra.

Now, let us see the effects of correlated disorder and hopping dimerization together in an AAH SSH ring. For that, we begin our discussion with Fig. 3, where the behavior of individual energy eigenvalues with flux $\phi$ is shown. The results are obtained for the non-staggered and staggered cases under two different conditions of intra- and inter-cell hopping integrals. Several interesting features are obtained those are analyzed one by one as follows. Firstly, all the energy levels are non-degenerate and they are periodic in $\phi$ showing $\phi _{0}$ flux-quantum periodicity. Secondly, the interplay between the dimerized hopping integrals and the AAH modulation strongly depends on the limiting condition i.e., whether $t_{1}$ is less or greater than $t_{2}$. In both these limiting conditions, two different kinds of energy levels are available. Few energy levels are almost insensitive to the flux $\phi$, those are referred to as ‘flat’ energy levels, and the rest of the other energy levels exhibit finite variation. A non-zero contribution in persistent current is obtained only from the later type of energy levels since the current is directly proportional to the variation of energy with $\phi$. The co-existence of zero and finite current carrying states are usually not available in conventional ring systems, and because of this mixing we can have the possibility of getting a transition from the high current carrying phase to the low one, and vice versa, depending on the electron filling. This behavior can be understood from our forthcoming discussion. Thirdly, the nature of the current carrying states situated towards the band center is strongly influenced by the intra- and inter-cell hopping integrals, both in the staggered and non-staggered cases, which we confirm through our exhaustive numerical calculations. We find that for the non-staggered ring, the slope of the current carrying states gets enhanced with increasing the correlated disorder strength *W* when $t_{1}>t_{2}$. A similar kind of behavior is also obtained for the staggered ring in the other limiting condition i.e., when $t_{1}<t_{2}$. Such a phenomenon is quite unusual since the common wisdom suggests that, the slope always decreases with increasing the impurity strength (yielding lesser current). For the non-staggered and staggered rings, the common picture (viz, lowering the slope) is obtained for the conditions $t_{1}<t_{2}$ and $t_{1}>t_{2}$, respectively. The key conclusion that we make from the energy-flux spectra is that the interplay between the dimerized hopping integrals and the sign of the site energies in each unit cell is highly important for determining the behavior of the individual energy levels.

### Current-flux characteristics

**Figure 4:**
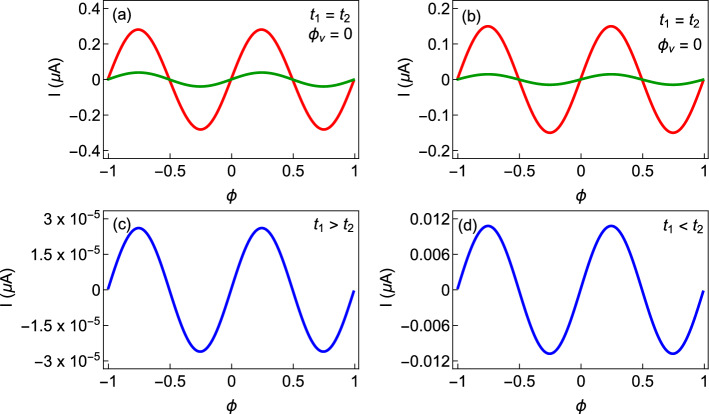
Current-flux characteristics in the half-filled limit where the first row is associated with the AAH ring (without any hopping dimerization, $t_{1}=t_{2}=1$), while the second row is for the SSH ring (without AAH modulation, $W=0$). For the AAH ring, the results for the non-staggered and staggered cases are shown in (**a**) and (**b**), respectively. On the other hand, for the SSH ring, we choose $t_{1}=1$ and $t_{2}=0.5$ in (**c**) and in (**d**) we take $t_{1}=1$ and $t_{2}=1.5$. The ring size $N=20$. In the first row, the red and green lines correspond to $W=1$ and 1.25, respectively.

The behavior of the persistent current as a function of flux $\phi$ for the cases considered in Fig. 2 is shown in Fig. 4 in the half-filled limit. Here a relatively bigger ring is taken into account such that the number of electrons becomes quite moderate in the limit of half-filling. In Fig. 4a and b, the currents are shown for two distinct AAH modulation strengths, namely, $W=1$ eV (red curve) and $W=1.25$ eV (green curve). We set $t_{1}=t_{2}=1$ eV for both the non-staggered ring (Fig. 4a) and the staggered ring (Fig. 4b) to capture only the essence of correlated disorder on persistent current. The usual reduction of current with impurity strength is obtained in Fig. 4a and b. The phase of the current (magnitude and sign) for a particular *W* and the reduction of current amplitude with the enhancement of *W* solely depend on the variation of ground state energy with respect to the AB flux $\phi$. The ground state energy, on the other hand, is directly linked with the distinct energy levels. When the slopes of the successive current-carrying energy levels are opposite, mutual cancellation takes place, resulting in a reduction of current. Usually, the net current comes from the energy levels close to the topmost filled level, as the contributions of the other energy levels are almost mutually cancelled from each other. Thus, the filling factor plays an important role, which can be understood more clearly from our subsequent analysis. For a particular filling, the suppression of current, on the other hand, occurs due to the enhanced electron scattering at different lattice sites with *W*. This reduction of current with increasing disorder strength is well studied in the literature^32^. To study the effect of hopping dimerization on persistent current, we consider SSH ring without any AAH disorder and the corresponding results for $t_{1}>t_{2}$ and $t_{1}<t_{2}$ are shown in Fig. 4c and d respectively. For $t_{1}>t_{2}$ case, the magnitude of current is vanishingly small, whereas for $t_{1}<t_{2}$ case, it is only a few nA. Overall, we observe that the non-staggered and staggered AAH rings are capable of producing large persistent current than the SSH rings. Now let us see their combined effect on the persistent current.

**Figure 5:**
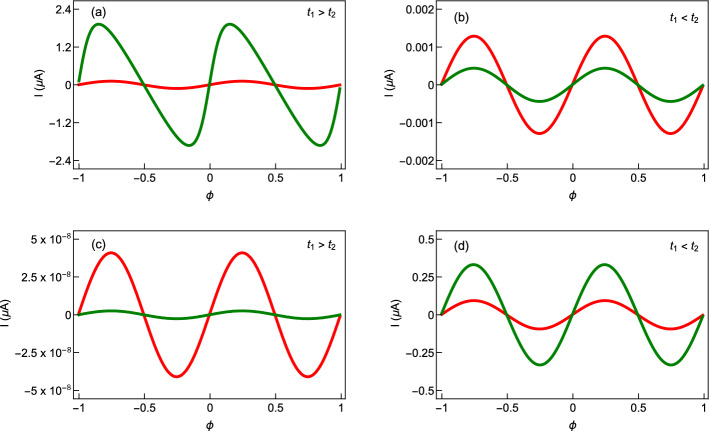
Current-flux characteristics for the AAH SSH rings in the half-filled band case at two different AAH modulation strengths, where the red and green curves correspond to $W=1$ and 1.25 eV, respectively. The first row represents the non-staggered [(**a**) $t_{1}>t_{2}$ and (**b**) $t_{1}<t_{2}$] case while the second row represents the staggered one [(**c**) $t_{1}>t_{2}$ and (**d**) $t_{1}<t_{2}$]. Here we choose $N=20$, and all other parameters are the same as given in Fig. 3.

From the behavior of the energy levels discussed in Fig. 3, it can be guessed that some anomalous features might be expected in the current-flux characteristics as well. Figure 5 displays the variation of persistent current with flux $\phi$, for the non-staggered and staggered AAH SSH rings under two different conditions of the hopping integrals. The currents are computed for the half-filled band case, considering $N=20$. In each of the spectra, the currents are shown for two distinct AAH modulation strengths those are $W=1$ eV (red curve) and $W=1.25$ eV (green curve). For the non-staggered ring with $t_{1}<t_{2}$ and for the staggered ring with $t_{1}>t_{2}$, the usual reduction of current with impurity strength is obtained (Fig. 5b,c). Moreover, in these two cases, the current amplitude is also vanishingly small. But, unconventional behavior occurs in these AAH SSH rings when we change the condition of hopping dimerization, as clearly reflected from the spectra given in Fig. 5a and d. The current amplitude gets *enhanced* with increasing the impurity strength *W*. The underlying mechanism relies on the specific correlation of the AAH modulation, staggered and non-staggered conditions, and hopping dimerization. This gives a clear indication of transition from a low conducting phase to a high conducting one. This is an interesting finding of our analysis.

### Variation of current with AAH modulation strength *W*

**Figure 6:**
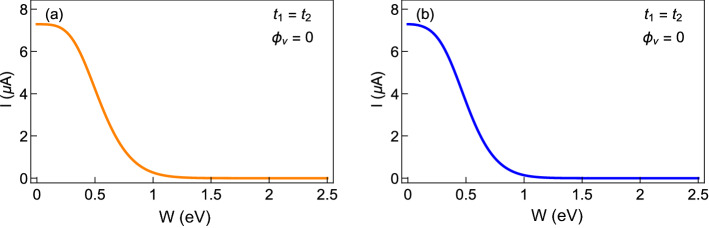
Magnitude of current, at a typical flux $\phi =0.2$, with disorder strength *W* at half-filling for the AAH ring (in the absence of any hopping dimerization, $t_{1}=t_{2}=1$) with $N=20$. Here (**a**) and (**b**) represent the non-staggered and staggered rings, respectively.

With the hint of getting a transition from a low conducting phase to high conducting one under certain physical conditions, it is indeed required a thorough checking of the dependence of current with the disorder strength. Before we do that for AAH SSH rings, we plot the behavior of persistent current as a function of AAH modulation strength (absence of hopping dimerization) for non-staggered and staggered AAH rings as shown in Fig. 6a and b, respectively. Here the flux $\phi$ is fixed at $\phi =0.2$ and $N=20$. As we observed in Fig. 4a and b, the usual reduction of current with disorder strength is clearly visible in Fig. 6a and b. For low values of *W*, the current is a few $\upmu$A, decreases with *W* and beyond $W\sim 1$, the current seems to be vanishingly small, and falls off to zero.

**Figure 7:**
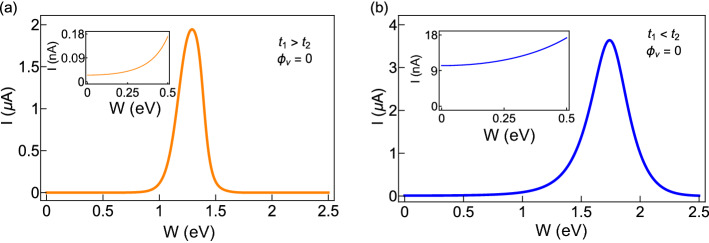
Same as Fig. 6, in presence of the hopping dimerization (viz, for the AAH SSH ring), where (**a**) $t_{1}=1$, $t_{2}=0.5$ and (**b**) $t_{1}=1$ and $t_{2}=1.5$. Insets are included to show that current is finite at low disorder strengths.

Next, we choose those two cases in which the unusual feature is seen in Fig. 5 and plot the current amplitudes in Fig. 7 at a typical flux by varying the AAH modulation strength *W* in a wide range. As we primarily focus on the variation of current amplitude with disorder strength, we plot the absolute value of the current with *W*. Both for the non-staggered and staggered cases, we find that beyond a critical disorder strength, the current amplitude gets enhanced significantly, and after reaching a maximum it decreases and eventually drops to zero for large enough *W*. From the variation of current with *W*, the transition from a low conducting phase to high conducting one is clearly seen, and it is purely due to the specific correlation in site energies and the hopping dimerization. Here we would like to mention one point, we have not found any compact analytical expression of the critical disorder strength where the current gets a maximum. Our detailed numerical calculations suggest that this critical disorder strength strongly depends on the choices of intra- and inter-cell hopping integrals and electron filling factor. Further studies can be done to have an analytical expression for the critical disorder strength. Moreover, from the insets of Fig. 7a and b, we confirm that the current at $W=0$ in very small, of the order of a few nA or even than an nA, but it is not zero. Such a small persistent current is completely due to the hopping dimerization of the AAH SSH ring.

### Role of AAH phase on current

**Figure 8:**
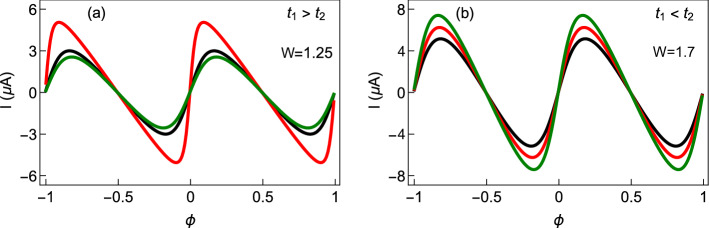
Persistent current as a function of AB flux $\phi$ at different values of $\phi _{\nu }$ for (**a**) non-staggered and (**b**) staggered AAH SSH rings, in the half-filled band case, considering $N=12$. In each plot, the black, red, and green colors are associated with $\phi _{\nu }=0$, $\pi /3$, and $\pi /2$, respectively.

In this sub-section, we investigate the role of AAH phase factor $\phi _{\nu }$ on circular current. Figure 8 displays the variations of persistent current as a function of AB flux $\phi$ at three typical values of $\phi _{\nu }$, those are represented by three different colored curves. Both for the staggered and non-staggered cases, a finite change in current is obtained with the modification of the phase factor $\phi _{\nu }$. This is expected as the site energies of the ring get modified with the change of $\phi _{\nu }$, which in turn, alters the energy eigenvalues and thus the current.

**Figure 9:**
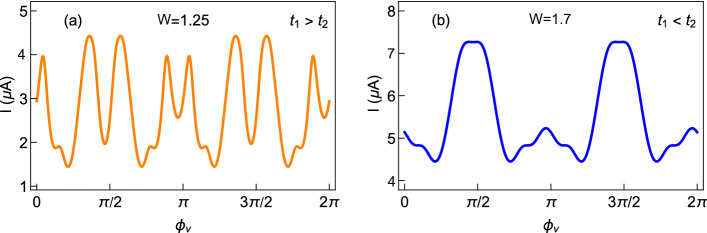
Absolute current at a particular flux $\phi$ ($\phi =0.2$) with respect to the AAH phase factor $\phi _{\nu }$ for the AAH SSH ring. The ring size, filling factor, and the meaning of (**a**) and (**b**) are the same as mentioned in Fig. 8.

To see the response for any other values of $\phi _{\nu }$, in Fig. 9, we plot the variation of current amplitude, determined at at a typical magnetic flux $\phi =0.2$, as a function of $\phi _{\nu }$ by varying it continuously in a wide range. The results are quite interesting. Both for the staggered and non-staggered rings, an oscillatory pattern with large peaks and dips is obtained. It gives a clear sign that the current amplitude and conducting behavior can be regulated selectively by means of $\phi _{\nu }$ without altering any other physical parameters describing the system.

### Dependence of current on $N_{e}$

**Figure 10:**
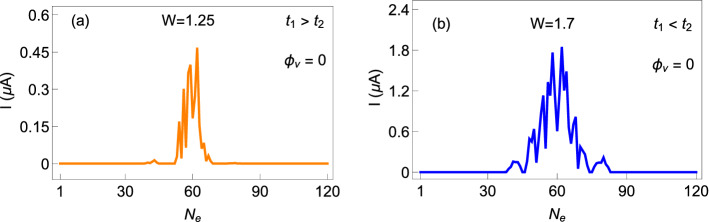
Absolute current as a function of total number of electrons $N_{e}$ at $\phi =0.2$ for (**a**) non-staggered and (**b**) staggered AAH SSH rings. Here we set $N=60$.

To inspect the dependence of circular current on the total number of electrons $N_{e}$ in the ring system, in Fig. 10 we plot the absolute current at a particular flux by varying $N_{e}$ in a wide range. Both the non-staggered and staggered rings are taken into account, like previous figures, and currents are computed at $\phi =0.2$ for each $N_{e}$, considering a relatively bigger ring with $N=60$. For smaller and higher numbers of electrons, currents are vanishingly small, while appreciable current is obtained for a small window around the half-filling. This is completely in contrast to a perfect ring or an uncorrelated/correlated disordered ring^41,57^, and occurs due to the presence of ‘flat’ energy levels along with the levels possessing finite slopes (can be seen clearly from the energy-flux spectra given in Fig. 3). Thus, electron filling has an important role to have a non-zero current.

### Effect of system size

**Figure 11:**
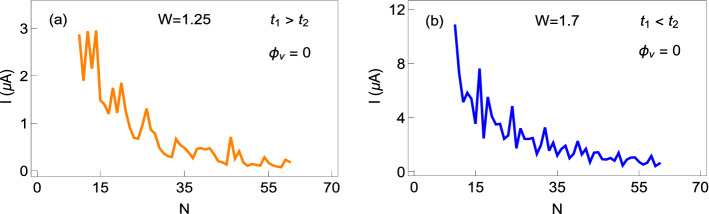
Absolute current as a function of the ring size in the half-filled limit for the (**a**) non-staggered and (**b**) staggered AAH SSH rings.

As persistent current is a mesoscopic phenomenon, it is relevant to check how the current varies with the ring size. In Fig. 11 the results are shown, where the currents are computed at a typical flux $\phi =0.2$, like in previous cases, both for the non-staggered and staggered half-filled rings. The overall envelope shows a decreasing nature, as expected, but for some particular cases, current shows an increasing behavior with the ring size. This oscillating tendency with system size is the generic feature in mesoscale regime and it is directly linked with the quantum interference of electronic waves. The nature of the envelope also depends on the filling factor, but the fact is that, irrespective of the electron filling, the current eventually reduces to zero when the ring size becomes too large.

### Co-existence of conducting and non-conducting states

**Figure 12:**
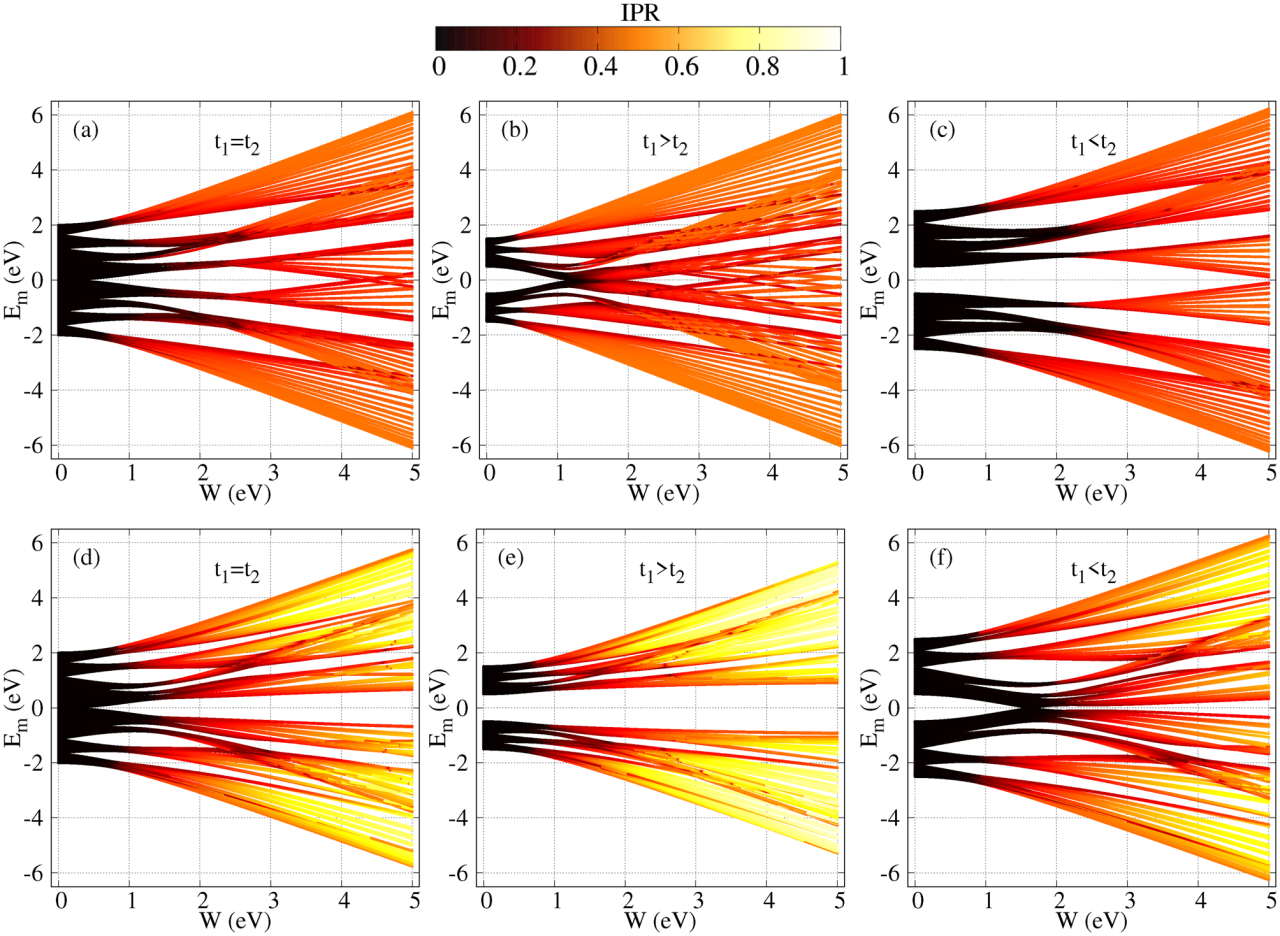
Density plot of energy eigenvalues and the IPRs of different energy eigenstates with disorder strength *W*, where the first and second rows are associated with the non-staggered and staggered conditions, respectively. The first column shows the results only for the AAH ring ($t_{1}=t_{2}=1$), whereas the rest of the spectra are associated with the AAH SSH ring under different conditions ($t_{1}>t_{2}$ and $t_{1}<t_{2}$). The results are worked out for $N=60$ and $\phi =0.2$.

The co-existence of conducting and non-conducting states is re-checked in Fig. 12 in another way by calculating inverse participation ratios of distinct energy eigenstates. Each spectrum of Fig. 12 represents the density plot, where the variations of energy eigenvalues and IPRs are shown as a function of the AAH modulation strength *W*, both for the staggered and non-staggered AAH SSH rings, under three different hopping conditions, namely, $t_{1}=t_{2}$, $t_{1}>t_{2}$, and $t_{1}<t_{2}$. Several fascinating results are obtained from the density spectra. The first observation is that for the non-staggered ring with $t_{1}<t_{2}$ (Fig. 12c) and the staggered ring with $t_{1}>t_{2}$ (Fig. 12e), a wide gap around $E=0$ persists between the two sub-bands, where each of these sub-band contains other multiple narrow bands with smaller gaps. The arrangement of the energy levels into two bigger bands is associated with the hopping dimerization, while in each sub-band the appearance of mini bands with smaller gaps is involved with the cosine modulation. The two bigger energy bands merge at a particular modulation strength for the cases with $t_{1}>t_{2}$ and $t_{1}<t_{2}$, as clearly seen from the plots given in Fig. 12b and f. Note that these two cases, apart from the ring size, gave rise to anomalous behavior, that is current increases with increasing disorder strength (see Fig. 7). This gap closing plays a vital role to have an anomalous signature in the current-flux spectra that we observe in Fig. 7a and d. However, for the cases with $t_{1}=t_{2}$, there is no gap around $E=0$ for the lower values of *W* (Fig. 12a,d). For the non-staggered case (Fig. 12a), a small gap opens around $E=0$, but eventually, at large disorder strengths, a three-band spectrum is observed, which is the usual AAH effect. On the other hand, for the staggered case with $t_{1}=t_{2}$, once the gap opens for a particular *W*, that gap persists throughout the given *W*-range (Fig. 12d). In the same contexts, it should be noted here that we observe additional gap closings at $W\sim 4$ eV in Fig. 12a, $W>3.5$ eV in Fig. 12b, and $W\sim 5$ eV in Fig. 12c. However, all the states near those gap closings are highly localized and no current carrying states thus exist. Therefore, near those additional gap closings, no further current enhancement will occur. The second important phenomenon that is seen in Fig. 12 is that, unlike a regular diagonal AAH system, all the energy states do not get localized beyond a critical disorder strength. It is well known that in the absence of hopping dimerization and without any staggered scenario, all the states get localized beyond the critical disorder strength 2*t* (if $t_{1}$ and $t_{2}$ are set to *t*). But for our dimerized AAH system, we can clearly see that the extended and localized states co-exist. Moreover, this co-existence occurs at multiple energies. So, naturally, we have the possibility to get energy-dependent mobility edges. This is indeed an interesting finding.

### Interplay of random disorder and hopping dimerization

In general, random (uncorrelated) disorders have much stronger localization behavior than correlated ones. However, for the sake of completeness, we compare our results with random disordered rings. To keep the true nature of random disorder, the staggered condition on the site potentials are excluded. Particularly, we consider three different hopping conditions, namely $t_{1}=t_{2}$, $t_{1}>t_{2}$, and $t_{1}<t_{2}$. The first condition corresponds to a randomly disordered ring without any hopping dimerization, and the other two denote SSH rings with random disorder. The random on-site potentials are included in site energies ‘randomly’ from a Box distribution function of width 2*W* to be consistent with the notion of AAH modulation strength. All the results are obtained by averaging over 1000 distinct random configurations.

**Figure 13:**
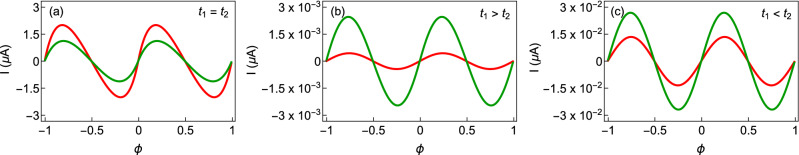
Current-flux characteristics for random disordered rings in the half-filled band case at two different disorder strengths, where the red and green curves correspond to $W=1$ and 1.25 eV, respectively. (**a**) $t_{1}=t_{2}=1$, (**b**) $t_{1}=1$, $t_{2}=0.5$, and (**c**) $t_{1}=1$, $t_{2}=1.5$. The ring size $N=20$. The currents are averaged over 1000 distinct random configurations.

The behavior of the persistent current as a function of flux $\phi$ for $t_{1}=t_{2}$, $t_{1}>t_{2}$, and $t_{1}<t_{2}$ are shown in Fig. 13a–c, respectively. Like before, two different random disorder strengths are considered, namely $W=1$ (red curves) and $W=1.25$ (green curves) and for the case of half-filled. In the absence of hopping dimerization, the usual reduction in the current is observed. For $t_{1}\ne t_{2}$ cases (Fig. 13b,c), the magnitudes of currents are relatively small compared to the $t_{1}=t_{2}$ case. But interestingly, unconventional behavior occurs, as we observed in the AAH SSH rings (Fig. 4a,d). In the latter two cases, the current amplitude increases with the random disorder strength *W*. Now, here we do not have any staggered scenario. And it is also known that all the energy eigenstates are localized irrespective of the random disorder strength. Therefore, it is the hopping dimerization that prevents such localization in presence of random disorder. Thus, the hopping dimerization plays a key role in the enhancement of current with increasing the disorder strength both in the correlated and uncorrelated disordered cases.

**Figure 14:**
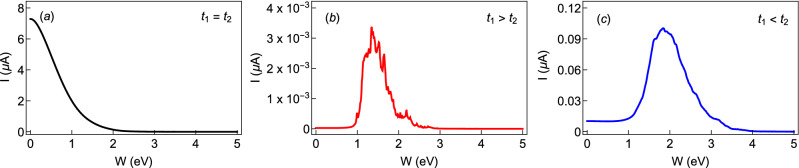
Magnitude of current, at a typical flux $\phi =0.2$, with random disorder strength in the half-filled band case. (**a**) $t_{1}=t_{2}=1$, (**b**) $t_{1}=1$, $t_{2}=0.5$, and (**c**) $t_{1}=1$, $t_{2}=1.5$. The ring size $N=20$. The currents are averaged over 1000 distinct random configurations.

In Fig. 14, the persistent current is plotted as a function of random disorder strength *W* for a fixed AB flux $\phi =0.2$, for three different conditions of $t_{1}$ and $t_{2}$, like previous cases. As expected, the current decreases with *W* for $t_{1}=t_{2}$ as shown in Fig. 14a. This is consistent with our previous results for AAH rings in the absence of hopping dimerization (Fig. 4). For $t_{1} > t_{2}$ (Fig. 14b), the current magnitude is relatively less compared to the case of $t_{1}<t_{2}$ (Fig. 14b). However, in both cases, the current is vanishingly small for low values of *W*, then it increases with increasing *W*, becomes maximum and again falls off to zero for large disorder strengths as it should be. It is also important to note that the current magnitudes of the AAH SSH is observed to be higher than the random disordered cases. This is because for the random (uncorrelated) disordered cases, we average the currents over a large number of configurations, but for the correlated case, it is just a single configuration. Because of the averaging, there is more cancellation of currents due to the many negative slopes and hence the current magnitude is less for the random disordered cases.

*Experimental feasibility*: There are several proposals to realize a ring-like geometry^58–62^. To realize an SSH ring, the different hopping integral values can be achieved either by using two different atoms in the successive sites^63^ or by arranging the atoms with different spacing in the successive bonds^64,65^. The distance between two atoms can be manipulated with desired precision via the atom manipulation technique^66^. To achieve the AAH profile in a ring, two counter-propagating laser beams having wave vectors $k_{1}$ and $k_{2}$ are used^67^, where the incommensuration parameter is defined by the factor $k_{1}/k_{2}$. Once the potential profile is formed, the atoms are trapped in those profiles and one can get an AAH ring. Together with these proposals and possibilities of realizing an SSH ring and an AAH ring, one may construct an AAH SSH ring experimentally.

## Closing remarks

In the present work, we have essentially focused on the combined effects of hopping dimerization and cosine modulation in site energies, on magnetic response in an isolated AB ring. Depending on the allocation of site energies, two different kinds of ring systems have been taken into account those are referred to as non-staggered and staggered ones. Employing a tight-binding framework, we have determined energy eigenvalues, flux-driven circular current, and the localization behavior of different energy eigenstates under different input conditions. The random disordered scenario has also been discussed to compare the results with the correlated ones. The key findings of our analysis are as follows.

- Co-existence of ‘flat’ energy levels along with the current carrying states. Due to this mixing of zero and non-zero current carrying states, we get a switching from low to high current carrying phase and vice versa. Such a behavior is no longer obtained in traditional AB rings.
- Unlike the conventional disordered rings, here we have found current enhancement with increasing the impurity strength, which is a transition from a low conducting phase to a high conducting one.
- The current amplitude can be tuned selectively by means of the AAH phase $\phi _{\nu }$.
- The electron filling factor plays an important role to have a net current, due to the existence of flat energy levels and energy levels with finite slopes. Across the half-filled band case, we get a large response.
- Current is also sensitive to the ring size, as it is a mesoscopic phenomenon. The current amplitude decreases with increasing the size of the ring, and for a large enough ring it reaches the vanishing limit.
- The density plot of IPRs clearly confirms the co-existence of the localized and delocalized energy levels. Energy-dependent mobility edges are also found.
- Like the correlated disordered cases, we have also found current enhancement with increasing the random disorder strength.
- The hopping dimerization is the key factor for the current enhancement with increasing the disorder strength, irrespective of the nature of the disorder, that is, uncorrelated or correlated.

Our analysis gives a suitable hint that the positional correlation among the constituent lattice sites along with the hopping dimerization plays an important role to have non-trivial signatures in electronic properties, and can be utilized in different sub-disciplines. Here we have provided one aspect by studying the magnetic response. Further studies can definitely be done in other contexts, and interesting phenomena might be expected.

## Data Availability

Derived data supporting the findings of this study are available from the corresponding author on request.
